# Bone Circulatory Disturbances in the Development of Spontaneous Bacterial Chondronecrosis with Osteomyelitis: A Translational Model for the Pathogenesis of Femoral Head Necrosis

**DOI:** 10.3389/fendo.2012.00183

**Published:** 2013-01-22

**Authors:** Robert F. Wideman, Rhonda D. Prisby

**Affiliations:** ^1^Center of Excellence for Poultry Science, University of ArkansasFayetteville, AR, USA; ^2^Department of Kinesiology and Applied Physiology, University of DelawareNewark, DE, USA

**Keywords:** osteomyelitis, femoral head necrosis

## Abstract

This review provides a comprehensive overview of the vascularization of the avian growth plate and its subsequent role in the pathogenesis of bacterial chondronecrosis with osteomyelitis (BCO, femoral head necrosis). BCO sporadically causes high incidences of lameness in rapidly growing broiler (meat-type) chickens. BCO is believed to be initiated by micro-trauma to poorly mineralized columns of cartilage cells in the proximal growth plates of the leg bones, followed by colonization by hematogenously distributed opportunistic bacteria. Inadequate blood flow to the growth plate, vascular occlusion, and structural limitations of the microvasculature all have been implicated in the pathogenesis of BCO. Treatment strategies have been difficult to investigate because under normal conditions the incidence of BCO typically is low and sporadic. Rearing broilers on wire flooring triggers the spontaneous development of high incidences of lameness attributable to pathognomonic BCO lesions. Wire flooring imposes persistent footing instability and is thought to accelerate the development of BCO by amplifying the torque and shear stress imposed on susceptible leg joints. Wire flooring *per se* also constitutes a significant chronic stressor that promotes bacterial proliferation attributed to stress-mediated immunosuppression. Indeed, dexamethasone-mediated immunosuppression causes broilers to develop lameness primarily associated with avascular necrosis and BCO. Prophylactic probiotic administration consistently reduces the incidence of lameness in broilers reared on wire flooring, presumably by reducing bacterial translocation from the gastrointestinal tract that likely contributes to hematogenous infection of the leg bones. The pathogenesis of BCO in broilers is directly relevant to osteomyelitis in growing children, as well as to avascular femoral head necrosis in adults. Our new model for reliably triggering spontaneous osteomyelitis in large numbers of animals represents an important opportunity to conduct translational research focused on developing effective prophylactic and therapeutic treatments.

## Introduction

**Figure 1:**
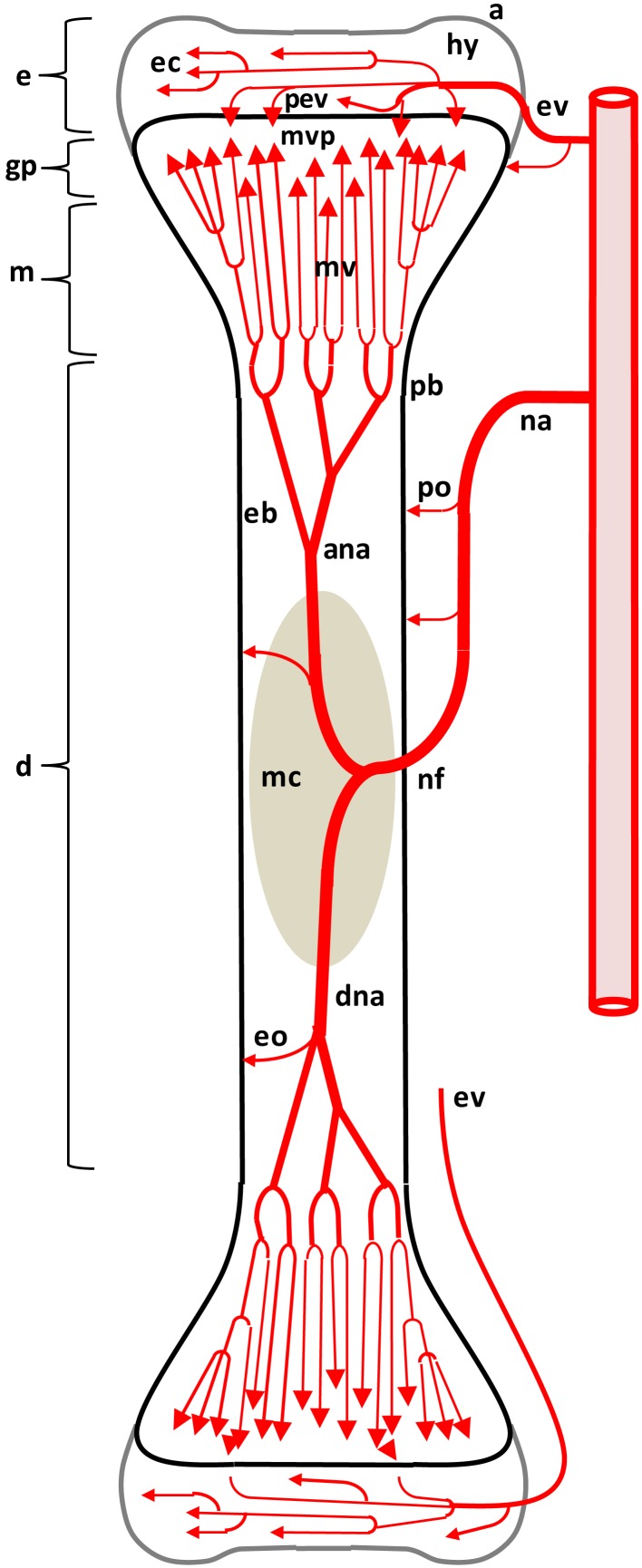
**Diagram depicting the arterial supply to a growing leg bone**. The epiphyseal vascular supply (ev) courses through epiphyseal vascular canals (ec) within the hyaline zone (hy) of the epiphysis (e). Terminal branches of some ev form penetrating epiphyseal vessels (pev) that supply the epiphyseal side of the growth plate (gp) or physis. The nutrient artery (na) penetrates the diaphysis (d) at the nutrient foramen (nf) and divides into ascending and descending branches (ana, dna) that subdivide repeatedly to form metaphyseal vessels (mv) within the metaphysis (m). The mv supply the metaphyseal vascular plexus (mvp) on the metaphyseal side of the gp. Additional features include: a, articular zone of epiphyseal cartilage; eb, endosteal bone; eo, endosteal arterioles; mc, medullary cavity; pb, periosteal bone; po, periosteal arterioles. Micro-anatomical terminology is consistent with the established anatomical nomenclature for avian bones (Beaumont, 1967; Lutfi, 1970a,b; Wise and Jennings, 1973; Howlett, 1979, 1980; Hunt et al., 1979; Duff, 1984a; Howlett et al., 1984; Thorp, 1986, 1988a; Ali, 1992; Farquharson and Jefferies, 2000).

**Figure 2:**
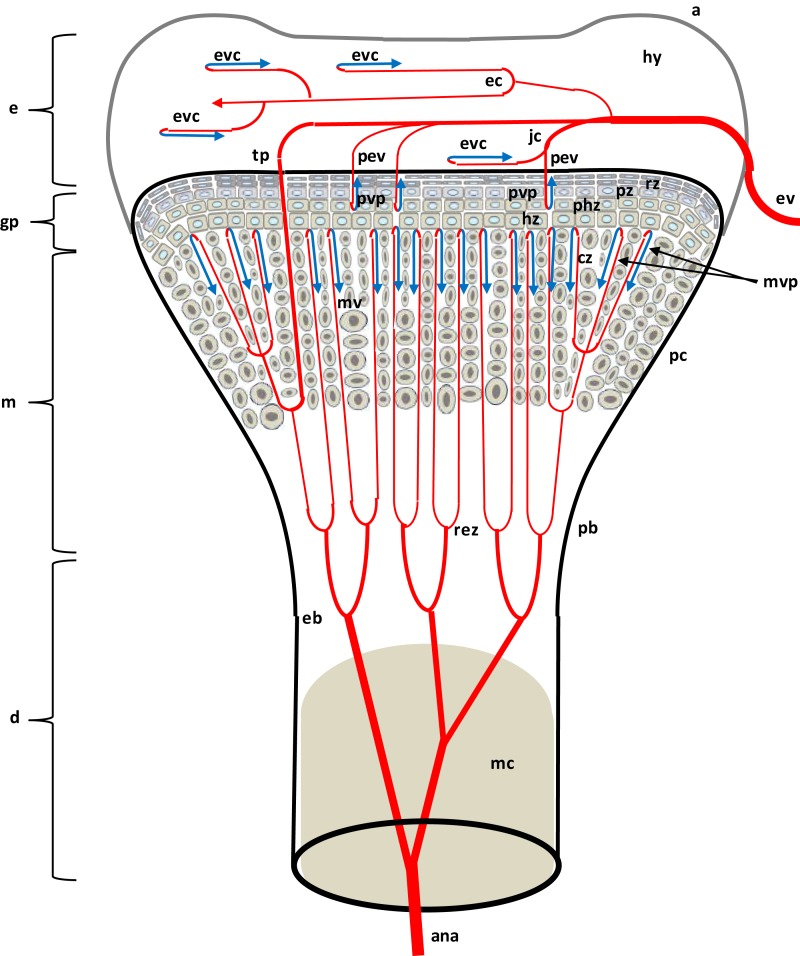
**Diagram depicting the proximal head of a growing leg bone**. The epiphyseal vascular supply (ev) courses through epiphyseal vascular canals (ec) within the hyaline zone (hy) of the epiphysis (e), or through junctional canals (jc) directed toward the growth plate (gp) or physis. Branches of the ev terminate as epiphyseal vascular capillary complexes (evc) within the hyaline zone, or become penetrating epiphyseal vessels (pev) that terminate as a penetrating vascular capillary plexus (pvp) supplying the resting zone (rz), proliferating zone (pz), and prehypertrophic zone (phz, also known as the maturing zone) of the gp. The ascending branch of the nutrient artery (ana) divides repeatedly inside the diaphysis (d) to form metaphyseal vessels (mv) within the metaphysis (m). The mv terminate as the metaphyseal vascular capillary plexus (mvp) supplying the calcifying zone (cz, also known as the degenerating hypertrophic zone) of the metaphysis. The hypertrophic zone (hz) normally is not penetrated by the pvp or mvp, but may rarely be penetrated by transphyseal vessels (tp). Additional features include: a articular zone of epiphyseal cartilage; eb, endosteal bone; mc, medullary cavity; pb, periosteal bone; and rez, resorption zone of the metaphysis. Micro-anatomical terminology is consistent with the established anatomical nomenclature for avian bones (Beaumont, 1967; Lutfi, 1970a,b; Wise and Jennings, 1973; Howlett, 1979, 1980; Hunt et al., 1979; Duff, 1984a; Howlett et al., 1984; Thorp, 1986, 1988a; Ali, 1992; Farquharson and Jefferies, 2000).

**Figure 3:**
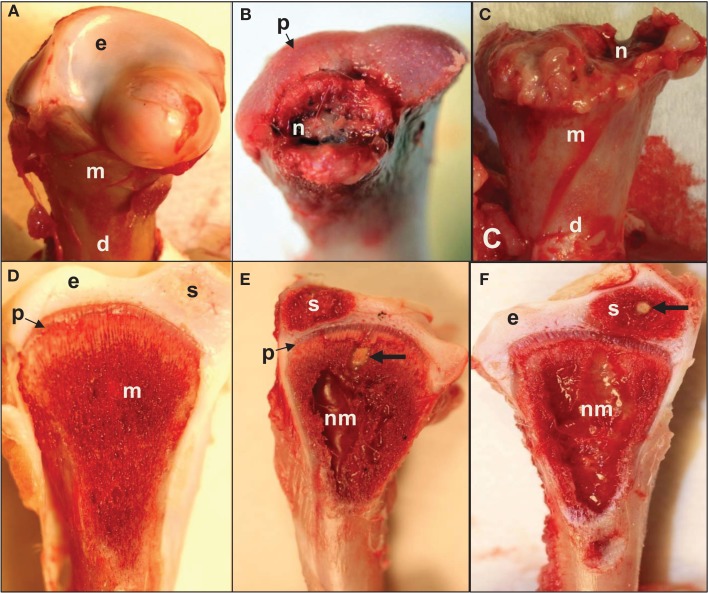
**(A)** Normal proximal femoral head with white cap of epiphyseal cartilage (e) on the metaphysis (m) above the diaphysis (d). **(B)** Epiphysis remained in the socket when the femur was removed from its socket (epiphyseolysis), revealing the surface of the growth plate (p) and bacterial necrosis (n) underlying the fractured remnant of the femoral head. **(C)** Femoral head fractured along the metaphyseal (m) plane to reveal widespread necrosis (n) within the shaft of the diaphysis (d). **(D)** In normal proximal tibiae, struts of trabecular bone in the metaphysis (m) transmit structural support from the diaphysis to the growth plate (physis, p) and articular cartilage (epiphysis, e). A secondary center of ossification (s) develops in the tibial epiphyseal cartilage of broilers after (approximately) day 28. **(E,F)** Large zones of necrosis have destroyed the central metaphysis (nm) and bacterial sequestrate (thick arrows) can occupy the calcifying zone beneath the growth plate as well as in the secondary center of ossification (s). Histologic examinations revealed microbial foci within the metaphyseal parenchyma adjacent to necrotic voids, in agreement with previous reports (Mutalib et al., 1983a; McNamee and Smyth, 2000). Voids undercutting the bony support structure facilitate micro-fracturing of the epiphyseal-physeal cartilage (Wideman et al., 2012).

Bacterial chondronecrosis with osteomyelitis (BCO, also known as femoral head necrosis) sporadically causes high incidences of lameness in meat-type chickens (broiler chickens, broilers). BCO has been diagnosed worldwide, and is considered the most common cause of lameness in commercial broilers (Pattison, 1992; McNamee et al., 1998; Butterworth, 1999; McNamee and Smyth, 2000; Bradshaw et al., 2002; Dinev, 2009). The pathogenesis leading to BCO is believed to be initiated by mechanical damage (e.g., osteochondrosis) to poorly mineralized columns of chondrocytes (cartilage cells) associated with the proximal growth plates of the rapidly growing femora and tibiae, followed by colonization of osteochondrotic clefts by hematogenously distributed opportunistic bacteria. Terminal BCO presents as necrotic degeneration and bacterial infection primarily within the proximal head (articular cartilage or epiphysis, growth plate or physis, and metaphysis; Figures 1 and 2) of the femur and tibiotarsus (hereafter referred to as the tibia), but also in the growth plates of other bones that are subjected to severe torque and shear stress, such as the fourth thoracic vertebrae (e.g., spondylopathy or spondylitis). The fourth thoracic vertebrae in birds articulate as the flexible pivot or fulcrum between the fused vertebrae of the notarium cranially and the bony pelvis caudally (Carnaghan, 1966; Wise, 1971; Nairn and Watson, 1972; Nairn, 1973; McCaskey et al., 1982; Kibenge et al., 1983; Mutalib et al., 1983a; Griffiths et al., 1984; Duff, 1990a; Pattison, 1992; Riddell, 1992; Thorp et al., 1993b; Thorp, 1994; Thorp and Waddington, 1997; McNamee et al., 1998; Butterworth, 1999; McNamee and Smyth, 2000; Bradshaw et al., 2002; Dinev, 2009; Stalker et al., 2010; Wideman et al., 2012). High incidences of both femoral and tibial BCO lesions (Figure 3) have been observed in lame broilers from commercial flocks.

In the absence of macroscopic lesions in the proximal femur, bacterial foci, and zones of necrosis typically are observed in the proximal tibia adjacent to the growth plate and within the metaphysis. The distal ends of the femora and tibiae rarely appear to be affected (Emslie et al., 1983; McNamee et al., 1999). Broilers from Scotland and Northern Ireland predominately exhibited BCO lesions in their proximal femora whereas broilers from Holland exhibited a predisposition for lesion development in the proximal tibiae (Thorp and Waddington, 1997). Previous reports also indicate that field outbreaks of BCO may affect only one leg while the contralateral leg appears macroscopically normal (McNamee et al., 1998; Dinev, 2009). Multiple opportunistic organisms have been isolated from BCO lesions, including *Staphylococcus aureus*, *Escherichia coli*, and *Enterococcus cecorum*, often in mixed cultures with other bacteria including *Salmonella* spp. (Nairn and Watson, 1972; Andreasen et al., 1993; Tate et al., 1993; Thorp et al., 1993b; McNamee et al., 1998; Butterworth, 1999; Smeltzer and Gillaspy, 2000; Joiner et al., 2005; Dinev, 2009; Stalker et al., 2010; Kense and Landman, 2011).

## Structural Immaturity of the Growth Plate

Modern broiler chicks weigh 40 g at hatch and are capable of growing to over 4 kg in 8 weeks. If humans grew at a similar rate, a 3-kg (6.6 lb) newborn baby would weigh 300 kg (660 lb) after 2 months. Doubling and re-doubling of the body mass almost seven times in 8 weeks cannot be sustained without equally dramatic increases in the size and structural integrity of the skeleton. Growth of the leg bones includes elongation accomplished via growth plates located at both ends of the shaft (diaphysis), as well as marked increases in the overall diameter attributable to highly dynamic remodeling of cortical bone (e.g., endosteal resorption in combination with periosteal formation). As demonstrated by Applegate and Lilburn (2002) a broiler’s femur increases from 2 cm in length on the day of hatch (day 1) to 7.6 cm in length by day 43, with the diameter at mid-shaft increasing from 2.5 to 9.4 mm during the same interval. The tibia increases from 2.9 to 10.9 cm in length between days 1 and 43, with the width at mid-shaft increasing from 1.9 to 9.4 mm. Femora and tibiae increase more than 70-fold in dry weight between days 1 and 43 (Applegate and Lilburn, 2002). Similarly dramatic estimates of rapid leg bone growth in broilers have been published by other investigators (Wise, 1970a,b; Riddell, 1975c; Thorp, 1988d; Bond et al., 1991; Leterrier et al., 1998; Williams et al., 2000a; Yalcin et al., 2001; Yair et al., 2012). The propensity for broiler strains to develop lameness when compared with laying strains of chickens was apparent more than 40 year ago and appears to be related to disproportions between a rapid early rate of body mass accretion vs. the progress of skeletal maturation rather than to relative differences in skeletal morphometrics or a caudal-to-cranial redistribution of muscle mass and thus the center of gravity (Wise, 1970a,b; Williams et al., 2000a). The highest incidences of lameness consistently occur in the fastest growing broiler flocks, and management strategies that tend to reduce the early growth rates also tend to reduce the incidence of skeletal disorders, lameness, and BCO (Riddell, 1983a,b; Classen, 1992; Robinson et al., 1992; Sorensen, 1992; Lilburn, 1994; McNamee et al., 1999; Bradshaw et al., 2002; Julian, 2005). These observations support a consensus hypothesis that the leg bones do not consistently mature rapidly enough to support the dramatic maximum growth potential of modern broilers (Wise, 1970b; LeBlanc et al., 1986; Classen and Riddell, 1989; Leterrier and Nys, 1992; Williams et al., 2000a, 2004).

**Figure 4:**
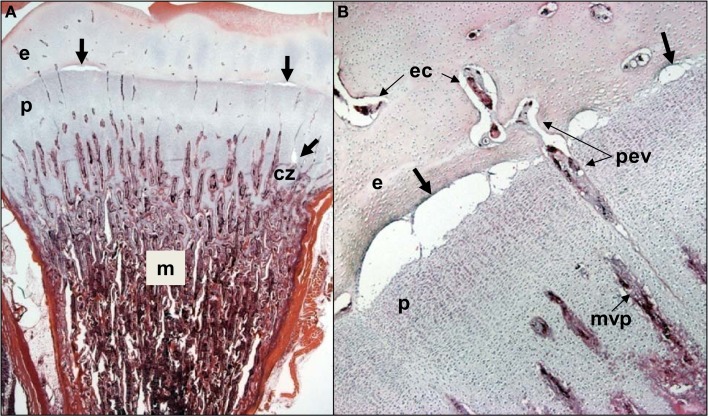
**(A)** Clinically healthy chicks were euthanized at 2 weeks of age, eviscerated and fixed by immersion in 10% buffered formalin for histological evaluation. Care was taken throughout to avoid pulling on or twisting the legs. After 5 days of fixation, the proximal half of each femur was removed with the pelvic socket still attached. The bones were decalcified, embedding in paraffin, sectioned at 5 μM thickness, and stained with hematoxylin and eosin. In two of the eight legs evaluated, narrow osteochondrotic clefts or voids (thick arrows) were evident at the boundary between the epiphysis (e) and the growth plate (physis, p) and occasionally between cartilage columns near the calcifying zone (cz) of the metaphysis (m). **(B)** Magnification of the clefts (thick arrows) at the junction of the epiphysis (e) and growth plate or physis (p). A penetrating epiphyseal vessel (pev) is threatened with transection. There is no evidence of inflammation or cellular debris adjacent to or within the clefts. The pre-mortem existence of these clefts cannot be proven, but clearly this key boundary zone is structurally fragile. Osteochondrotic clefts at the epiphyseal-physeal boundary and within the physeal cartilage are considered to predispose broilers to epiphyseolysis, which in turn is considered an early or initial macroscopic manifestation of BCO (Duff, 1984b, 1989a, 1990b; Duff and Randall, 1987; Thorp et al., 1993b; Bradshaw et al., 2002). Also shown are epiphyseal vascular canals (ec) and a metaphyseal vascular plexus (mvp).

Lameness in broilers rarely is attributable to a failure of cortical bone calcification or fracture of the diaphysis. Instead, the pathogenesis of BCO has been attributed to the presence of unusually long columns of chondrocytes within the proximal growth plate and adjacent metaphysis (Figure 2). When compared with mammalian growth plates, the avian growth plate is much thicker and the chondrocyte columns are aligned irregularly. These differences have been attributed to high longitudinal growth rates associated with very rapid growth plate turnover times in birds (estimated at 21 h) when compared with rats (4 days) and humans (20 days). Indeed, the rate of avian leg bone elongation is positively correlated with thickness of the growth plate, with the proximal ends of leg bones elongating twice as rapidly and having a thicker growth plate than the distal end of the same bone (Church and Johnson, 1964; Kember and Kirkwood, 1987; Leach and Gay, 1987; Thorp, 1988d; Kirkwood et al., 1989a,b; Kember et al., 1990; Hurwitz et al., 1992). Torque and shear forces chronically exerted at these key interfaces lead to micro-fracturing and the formation of osteochondrotic clefts within the epiphyseal and physeal cartilage layers (Figure 4; Wise, 1971; McCaskey et al., 1982; Riddell et al., 1983; Duff, 1984a,b,c, 1985, 1989a,b, 1990a,b; Julian, 1985; Duff and Randall, 1987; Thorp, 1988a,b,c, 1994; Thorp and Duff, 1988; Riddell, 1992; Thorp et al., 1993b; Thorp and Waddington, 1997; McNamee et al., 1998; Bradshaw et al., 2002; Dinev, 2009). Local biomechanical stresses and impaired blood flow to the epiphyseal-physeal cartilage contribute to the pathogenesis of osteochondrosis in a variety of animal species (Trueta and Amato, 1960; Riddell, 1975b; Boss and Misselevich, 2003; Ytrehus et al., 2004a,b,c, 2007). Osteochondrosis has been observed in the epiphyseal-physeal cartilage of leg bones and thoracic vertebrae of apparently healthy broilers exhibiting no symptoms of infectious or traumatic lameness or spondylolisthesis/spondylitis (Wise, 1971; McCaskey et al., 1982; Riddell et al., 1983; McNamee et al., 1998), suggesting lameness is not necessarily caused by direct mechanical damage *per se* but rather by an ensuing bacterial infection (McNamee et al., 1998).

## Vascular Supply to the Growth Plate

As diagrammed in Figures 1 and 2, the ends of growing avian leg bones include: (1) a cartilaginous epiphysis (e) comprised of articular (a) and hyaline (hy) cartilage; (2) the growth plate (gp) or physis having a cartilaginous matrix and long columns of chondrocytes in sequential maturational layers exhibiting distinctive phenotypes and biochemical characteristics, including progenitor or germinal chondrocytes (stem cells) of the resting zone (rz), the highly mitotic proliferating zone (pz), the prehypertrophic zone (phz), and the hypertrophic zone (hz); and (**3**) the metaphysis (m) consisting of calcifying (degenerating, apoptotic) chondrocytes and the newly formed osteoid of the calcifying zone (cz), spicules of trabecular bone that provide a support scaffolding for the growth plate (Figure 3D), and the resorption zone (rez) in which the trabecular bone thins and ultimately is resorbed to form the medullary cavity (mc) of the diaphysis (d; Figures 1 and 2; Wolbach and Hegsted, 1952; Lutfi, 1970a,b; Wise and Jennings, 1973; Bisaz et al., 1975; Riddell, 1975a; Carson et al., 1978; Poulos, 1978; Howlett, 1979; Hunt et al., 1979; Jande and Dickson, 1980; Huffer and Lacey, 1982; Lacey and Huffer, 1982; Duff, 1984a; Long et al., 1984a,b; Gay and Leach, 1987; Kember and Kirkwood, 1987; Kirkwood et al., 1989a,b; Kember et al., 1990; Pines and Hurwitz, 1991; Ali, 1992; Farquharson et al., 1992, 1996; Leach and Twal, 1994; Hatori et al., 1995; Roach et al., 1995; Farquharson and Jefferies, 2000).

The leg bones of fowl are supplied primarily by branches of the ischiadic artery, with a minor contribution from the external iliac artery (Nishida, 1963). Blood vessels enter the epiphysis at several sites (Howlett et al., 1984; Thorp, 1986, 1988a) and this epiphyseal vasculature (ev) subdivides into central arterioles coursing through blind-ended epiphyseal vascular canals (ec) within the hyaline zone of the epiphysis, or through junctional canals (jc) angled toward the growth plate (Figures 1 and 2). At the end of an epiphyseal vascular canal, an epiphyseal vascular complex (evc) is formed when the central arteriole divides into multiple capillary loops that make hairpin bends and re-converge as one or more venules carrying blood out of the canal. Arterioles within jc branch into penetrating epiphyseal vessels (pev) that descend through the epiphyseal-physeal boundary via regularly spaced perforating cartilage canals (not shown) to terminate as a penetrating vascular plexus (pvp) supplying the rz, pz and phz of the growth plate. The pvp consists of a central arteriole that divides into multiple capillaries making hairpin loops to re-converge as one or more venules carrying blood out of the perforating canal (Figures 2 and 4B). At the level of the rz and pz the capillary endothelium is fenestrated and exhibits a discontinuous basement membrane, whereas adjacent to the hz the endothelial cells are joined by tight junctions and surrounded by a continuous basement membrane (Beaumont, 1967; Lutfi, 1970b; Wise and Jennings, 1973; Hunt et al., 1979; Huffer and Lacey, 1982; Lacey and Huffer, 1982; LeBlanc et al., 1986; Thorp, 1986).

The metaphyseal vessels (mv) are terminal branches of the nutrient artery (na), which penetrates the diaphyseal cortex through the nutrient foramen (nf) located approximately at mid-shaft (Figures 1 and 2). Inside the diaphysis the ascending and descending branches of the nutrient artery (ana, dna) divide repeatedly to form mv that terminate in a metaphyseal vascular plexus (mvp) at the interface between the calcifying zone of the metaphysis and the hz of the growth plate. The mvp is formed by a central arteriole dividing into multiple capillaries having a fenestrated endothelium and forming hairpin loops that re-converge as venules exiting the calcifying zone of the metaphysis (Figures 2 and 4B; Beaumont, 1967; Lutfi, 1970b; Wise and Jennings, 1973; Poulos, 1978; Hunt et al., 1979; Howlett, 1980; Huffer and Lacey, 1982; Lacey and Huffer, 1982; Emslie and Nade, 1983, 1985; Howlett et al., 1984).

If the hz is viewed as a static stratum of chondrocytes occupying the interface between the physis and the calcifying zone of the metaphysis, then the hz would appear to be avascular because it apparently receives little or no blood flow from either the pvp or the mvp. Indeed, the zone of hypertrophy in birds has been defined as the level at which evc and mvp end as they approach the growth plate from opposite sides (Wise and Jennings, 1973; Huffer and Lacey, 1982; Lacey and Huffer, 1982; Long et al., 1984a). However, from a dynamic point of view the phenotype of each chondrocyte within the growth plate is ephemeral, existing only transiently as the bone lengthens. The pace of bone elongation is determined by the rate of mitosis within the pz which, as it thickens, pushes the physeal-epiphyseal boundary longitudinally. Each chondrocyte originating by mitosis at the boundary of the rz and pz remains approximately *in situ* or spatially fixed while sequentially passing through the prehypertrophic, hypertrophic, and calcifying phenotypes during the ensuing 21 h. This *in situ* synchronization of chondrocyte maturation creates the illusion that all zones of the growth plate are migrating together longitudinally (Lutfi, 1970a; Gay and Leach, 1987; Kember and Kirkwood, 1987; Kirkwood et al., 1989a,b; Kember et al., 1990; Pines and Hurwitz, 1991; Farquharson et al., 1992). A dynamic perspective on longitudinal bone growth provides compelling evidence that the perforating cartilage canals and associated pev must constantly be retreating, in concert with the longitudinally migrating physis and epiphysis. Indeed at the interface between the pz and phz, the retreating perforating cartilage canals are occluded by debris and fibrinoid remnants of the necrotic vasculature, and within the hz the compressed channels previously occupied by perforating canals are filled with scar tissue (Lutfi, 1970b; Hunt et al., 1979; Huffer and Lacey, 1982; Lacey and Huffer, 1982).

In contrast to the ongoing waves of occlusion and necrosis that destroy the retreating epiphyseal vessels, on the opposite side of the hz the ascending mv are simultaneously undergoing angiogenesis and elongation. The rapid advancement of the metaphyseal vasculature is causally synchronized with the longitudinal migration of the physeal-metaphyseal interface. Many of the invading mv advance through the preexisting scar tracts left behind by retreating pev, while other mv erode new channels through the hz (Lutfi, 1970b; Hunt et al., 1979; Lacey and Huffer, 1982; Thorp, 1986, 1988d). The advancing capillaries of the mvp form vascular buds that invade the lacunae of hypertrophic chondrocytes. Monocytes and phalanxes of chondroclasts (osteoclasts) accompany the invading vascular buds, perforating the chondrocyte lacunae, and eroding the adjacent cartilage matrix to form metaphyseal canals (Howlett, 1980). These canals serve as conduits for advancing mv to pursue the newly developing ranks of hypertrophic chondrocytes. As the leading edge of the metaphyseal vascular invasion migrates onward, most of the bypassed hypertrophic chondrocytes acquire a calcifying (degenerating, apoptotic) phenotype, and they are surrounded by osteoclasts and osteoid matrix. At the mid-metaphyseal level osteoblasts promote osteoid mineralization and the formation of closely packed spicules of trabecular bone. Near the diaphysis, osteoclastic activity erodes the trabecular bone and expands the metaphyseal canals into a latticework of broad interconnecting channels containing the mv and marrow cells (Wolbach and Hegsted, 1952; Leach and Nesheim, 1965; Lutfi, 1970b; Bisaz et al., 1975; Howlett, 1980; Lacey and Huffer, 1982; Pines and Hurwitz, 1991; Hurwitz et al., 1992; Hatori et al., 1995; Roach et al., 1995; Ohyama et al., 1997).

Transphyseal vessels (Figure 2) have been described penetrating through the entire growth plate to establish direct, patent communications or anastomoses between the epiphyseal and metaphyseal vascular beds (Westmoreland and Hoekstra, 1969; Nairn, 1973; Emslie and Nade, 1983; Emslie et al., 1984; Alderson et al., 1986a,b). Several investigators were unable to confirm the presence of transphyseal vessels (Beaumont, 1967; Lutfi, 1970b; Wise and Jennings, 1973; Poulos, 1978; Hunt et al., 1979; Howlett et al., 1984; Thorp, 1986). Putative transphyseal vessels have been reported in the leg bones of turkeys and broilers immediately post-hatch, when a cone of persistent embryonic cartilage extends from the epiphysis deep into the metaphysis (Lutfi, 1970b; Poulos, 1978; Thorp, 1986, 1988a,c). Some reports of transphyseal vessels in the differentiated avian growth plate may reflect a misinterpretation of the streaks of scar tissue left behind by receding pev. The advancing tips of mv appear to follow these remnant channels as the pathway of least resistance, with the compressed streaks again expanding at the terminus of retreating epiphyseal vessels, thereby providing the illusion of luminal continuity (Wise and Jennings, 1973; Hunt et al., 1979; Howlett et al., 1984; Thorp and Waddington, 1997). Nevertheless, evidence based on vascular infusion or filling with various substances, or serial sectioning, provides support for occasional transphyseal continuity across the avian growth plate, although in several cases the presumably patent lumen does resemble a dramatically narrowed and compressed remnant at the level of the hz (Alderson et al., 1986a; Thorp, 1988a,c). The direction of blood flow in putative transphyseal vessels and the nature of the arterial-venous communication at the physeal-metaphyseal interface vessels remains to be demonstrated.

The vascular relationships summarized above suggest the pev are responsible for providing nutritional support to the growth plate, whereas the advancing mv are responsible for its erosion and mineralization (Wise and Jennings, 1973). The causal relationship between growth plate dynamics, bone formation, and the microvascular architecture becomes readily apparent when the process is disturbed. For example, the formation of an avascular cartilage plug comprised of disorganized prehypertrophic chondrocytes (e.g., tibial dyschondroplasia) has been attributed to impaired mv penetration of the hz (Leach and Nesheim, 1965; Riddell, 1975a,b; Lacey and Huffer, 1982; Gay et al., 1985; Hargest et al., 1985; LeBlanc et al., 1986; Thorp et al., 1991, 1993a; Thorp, 1992; Farquharson et al., 1992; Parkinson and Thorp, 1996; Farquharson and Jefferies, 2000). In contrast, avian hypophosphatemic rickets is characterized by an exceptionally wide band of hypertrophic chondrocytes that do not acquire the calcifying (degenerative) phenotype in spite of extensive invasion by mv (Bisaz et al., 1975; Chan et al., 1977; Lacey and Huffer, 1982; Long et al., 1984a; Thorp, 1992; Thorp and Waddington, 1997). Finally, hypocalcemia caused by dietary calcium deficiency or Vitamin D_3_ deficiency is characterized by a widened pz containing proportionally elongated pev. The available evidence suggests hypocalcemia *per se* delays the necrosis of the pev and thereby delays the maturation of proliferating chondrocytes into prehypertrophic and hypertrophic chondrocytes. Absent a suitable availability of hypertrophic chondrocytes, metaphyseal vascular invasion of the hz is retarded and the calcifying zone narrows dramatically in hypocalcemic birds (Bélanger and Migicovsky, 1958, 1960; Bisaz et al., 1975; Jande and Dickson, 1980; Huffer and Lacey, 1982; Lacey and Huffer, 1982; Long et al., 1984b; Farquharson et al., 1993; Thorp and Waddington, 1997). The absolute levels, ranges or ratios of dietary calcium and inorganic or available phosphorus that contribute to these growth plate pathologies were summarized by Williams et al. (2000b).

## The Growth Plate Vascular Plexus and Susceptibility to Bacterial Infection

**Figure 5:**
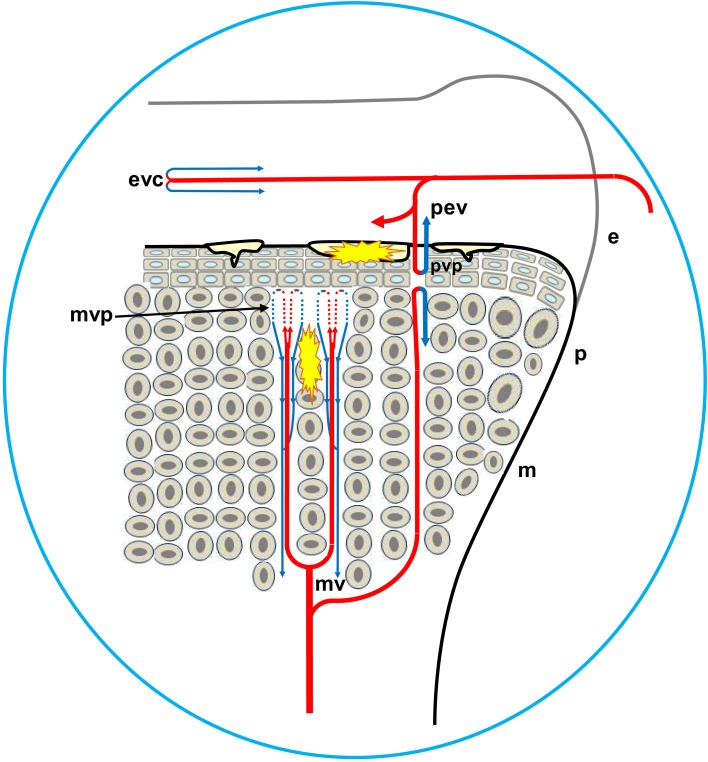
**Diagrammatic representation of the proximal head of the femur, depicting the formation of osteochondrotic clefts at the boundary between the epiphysis (e) and the growth plate (physis, p)**. Ascending metaphyseal vessels (mv) penetrate through canals between long columns of calcifying cells in the metaphysis (m). The metaphyseal vascular plexus (mvp) is formed by hairpin bends in fenestrated metaphyseal capillaries that return as venules coursing through the same canal (blue arrows). Translocated bacteria spread hematogenously and can exit the bloodstream through the fenestrated endothelium at the tips of the metaphyseal vascular plexus. The extravasated bacteria may adhere directly to the cartilage matrix, they colonize osteochondrotic clefts and zones of necrosis, and they form obstructive emboli in the metaphyseal vasculature. Similar hairpin bends and fenestrated capillary epithelia have been reported for the terminal epiphyseal vascular complex (evc), as well as for the terminus of penetrating epiphyseal vessels (pev) and the penetrating vascular plexus (pvp).

**Figure 6:**
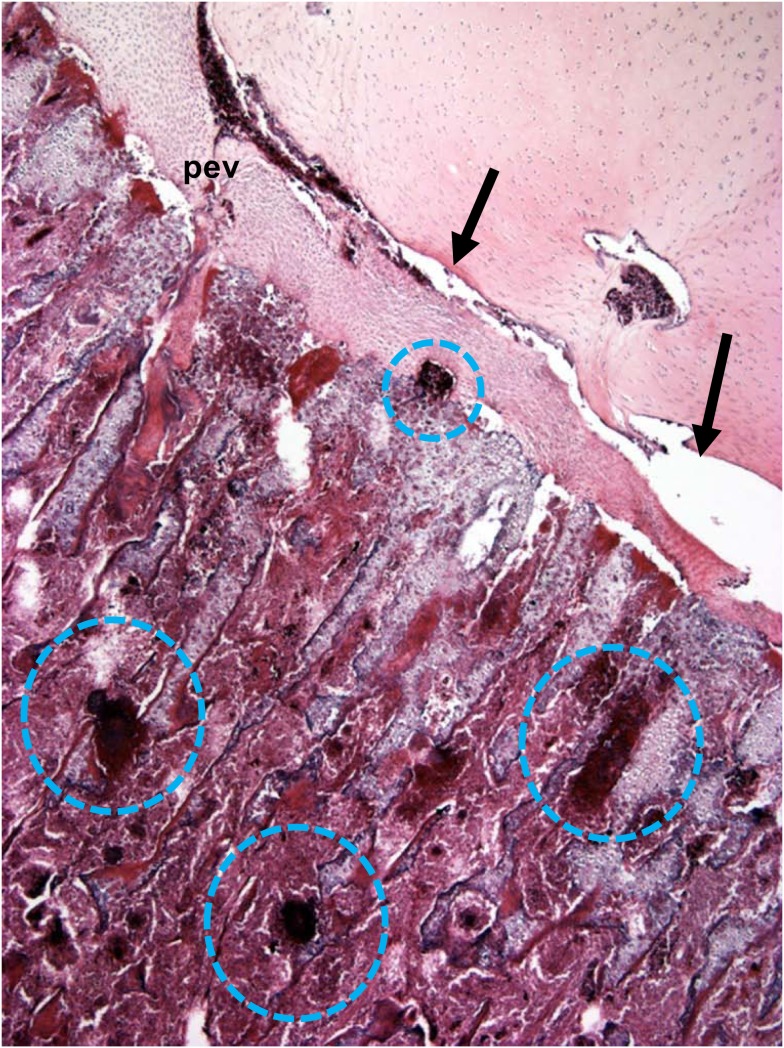
**Microphotograph of the proximal femoral head of a 38-day-old broiler with definitive femoral head necrosis (hematoxylin and eosin stain; 5 μm section)**. Bacterial foci are circled. Arrows indicate an infected osteochondrotic cleft at the interface between the epiphyseal cartilage and the physeal cartilage. A degenerating penetrating epiphyseal vessel (pev) is associated with the cleft.

Penetrating epiphyseal vessels and mv terminate on opposite sides of the growth plate by forming a pvp at the interface between the pz and phz, or a mvp at the interface between the cz and hz, respectively. The vascular plexus includes a tuft of capillaries possessing a fenestrated endothelium, with fenestrations large enough to permit cellular elements of the blood to pass into spaces within the cartilaginous matrix (Figures 2 and 5; Beaumont, 1967; Lutfi, 1970b; Hunt et al., 1979; Howlett, 1980; Emslie and Nade, 1983, 1985; Howlett et al., 1984). Bacteria transmitted to chicks from breeder parents, contaminated eggshells, or hatchery sources (Skeeles, 1997; McCullagh et al., 1998; Rodgers et al., 1999; McNamee and Smyth, 2000; Kense and Landman, 2011), or that enter the chick’s circulation via translocation through the integument, respiratory system, or gastrointestinal tract (Mutalib et al., 1983a,b; Andreasen et al., 1993; Thorp et al., 1993b; McNamee et al., 1999) spread hematogenously and can exit the bloodstream through the fenestrated endothelium at the tips of a vascular plexus on either side of the growth plate, or within the epiphyseal cartilage (Emslie and Nade, 1983, 1985). Hematogenously distributed bacteria possessing the specific ability to bind to bone collagen are significantly more virulent in their capacity to trigger osteomyelitis (Smeltzer and Gillaspy, 2000). The translocated bacteria adhere directly to the cartilage matrix, they colonize osteochondrotic clefts and zones of necrosis, and they form obstructive emboli in the metaphyseal vasculature. Neither cellular components of the immune system nor antibiotics appear capable of gaining access to established bacterial foci and sequestrate (Figures 5 and 6; Emslie and Nade, 1983; Emslie et al., 1983; Kibenge et al., 1983; Speers and Nade, 1985; Alderson et al., 1986a,b; Alderson and Nade, 1987; Thorp, 1988b; Thorp et al., 1993b; McNamee et al., 1998, 1999; McNamee and Smyth, 2000; Smeltzer and Gillaspy, 2000; Kense and Landman, 2011). Osteochondrotic clefts often truncate epiphyseal and metaphyseal blood vessels, contributing to focal ischemia, and necrosis. Local ischemia also has been attributed to sluggish blood flow and thrombosis caused by mechanical compression of the cartilage layers, the resting posture and inactivity of fully fed broilers, and an excessive resistance to flow through long, narrow metaphyseal vascular channels (Wise, 1971; McCaskey et al., 1982; Riddell et al., 1983; Duff, 1984a,b,c, 1985, 1989a,b, 1990a,b; Julian, 1985; Duff and Randall, 1987; Thorp, 1988b, 1994; Thorp and Duff, 1988; Riddell, 1992; Thorp et al., 1993b; Thorp and Waddington, 1997; McNamee et al., 1998; Bradshaw et al., 2002; Dinev, 2009). As the growth plates progressively migrate longitudinally with the ends of growing bones, previously formed bacterial sequestrate are left behind as focal zones of necrosis or large fibrinonecrotic abscesses in the metaphysis and diaphysis (Figures 3, 5, and 6; Emslie and Nade, 1983; Emslie et al., 1983, 1984; Daum et al., 1990; Thorp et al., 1993b; Skeeles, 1997; Wideman et al., 2012). Lytic substances released at sites of bacterial colonization promote generalized necrosis within the calcifying zone of the metaphysis, destroying the vasculature, and eliminating struts of trabecular bone that normally provide structural support to prevent micro-fracturing of the epiphyseal and physeal cartilage (Emslie and Nade, 1983; Emslie et al., 1984; Wyers et al., 1991; Wideman et al., 2012). Bacteria penetrating the epiphysis, perhaps via transphyseal vessels or perhaps directly via evc, trigger septic arthritis of the hock, and hip joints (Emslie et al., 1984; Emslie and Nade, 1985; Alderson et al., 1986b; Alderson and Nade, 1987; Thorp, 1988a; Daum et al., 1990; McNamee et al., 1998; Joiner et al., 2005).

## Experimental Models of BCO

**Figure 7:**
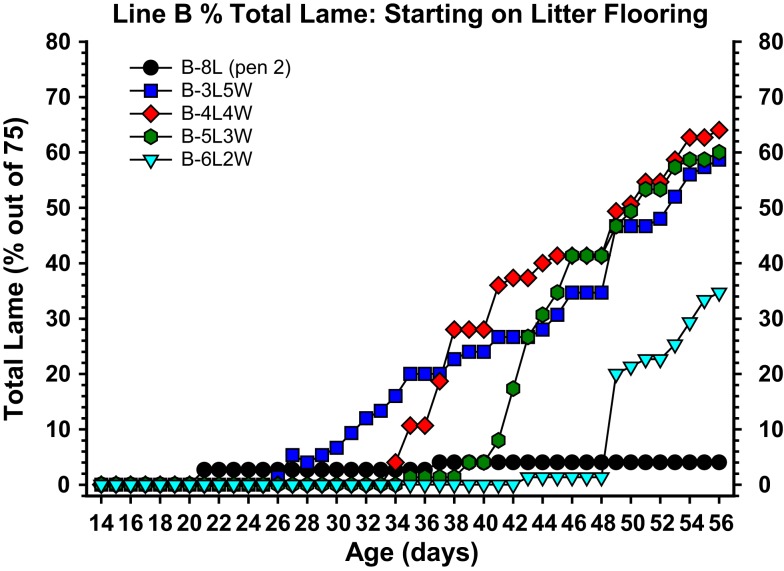
**Cumulative incidence of total lameness for broilers that at 1 day of age were placed on clean wood shavings floor litter (L), and either remained on litter for 8 weeks (8L) or were transferred from litter to flat wire flooring (W) for the remainder of the 8-week experiment, beginning at 3 weeks of age (3L5W), 4 weeks of age (4L4W), 5 weeks of age (5L3W), or 6 weeks of age (6L2W)**. All birds were pushed to grow as rapidly as possible to meet their genetic potential (23 h of light per day, high density feeds provided *ad libitum*, thermoneutral and well ventilated environmental conditions). The incidence of lameness remained typically low as long as the broilers remained on litter, however within 5–7 days after being transferred to wire flooring the incidence of lameness increased dramatically. Total lameness for the 3L5W, 4L4W, and 5L3W groups converged to between 59 and 64% by week 8.

The etiology, pathogenesis, and treatment strategies for BCO have been difficult to investigate because the incidence typically is low and sporadic in research flocks. Previously BCO has been reproduced by injecting broilers and turkeys intravenously with appropriate strains of *Staphylococcus* spp. in quantities sufficient to sustain bacteremia without triggering overt septicemia (Smith, 1954; Carnaghan, 1966; Wise, 1971; Nairn, 1973; Emslie and Nade, 1983, 1985; Emslie et al., 1983; Kibenge et al., 1983; Mutalib et al., 1983a; Griffiths et al., 1984; Alderson et al., 1986b; Daum et al., 1990). Experiments recently were designed with the objective of creating a research model for reliably triggering BCO without purposefully exposing the broilers to known pathogens. A wire flooring system was developed^1^ to create sustained footing instability and thereby exert persistent additional mechanical torque and shear stress on susceptible leg joints. Based on our current understanding, excessive shear stress causes micro-trauma and osteochondrosis of the epiphyseal-physeal cartilage, accompanied by mechanical truncation or thrombotic occlusion of metaphyseal blood vessels, and hematogenous colonization by translocated bacteria (McCaskey et al., 1982; Riddell et al., 1983; Duff, 1984b, 1989a,b; Emslie et al., 1984; Duff and Randall, 1987; Thorp and Duff, 1988; Hocking, 1992; Thorp et al., 1993b). Incidences of lameness between 20 and 60% are reliably induced by rearing fast growing broilers on wire flooring (Figure 7). The continuum of pathognomonic BCO lesion progression is readily apparent, including the consistent presence of proximal femoral head necrosis and proximal tibial head necrosis (Wideman et al., 2012). Most of the lameness develops between 6 and 8 week of age, as has been reported for field outbreaks of BCO (McNamee et al., 1998; Dinev, 2009). Lameness progresses very rapidly in broilers that appeared to be healthy during the preceding 24–48 h, as previously reported by Joiner et al. (2005). Males and females differ minimally in their susceptibility to lameness when reared together on wire flooring. Broilers completely immobilized by lameness typically exhibit the most severe BCO lesions, however the pathogenesis of BCO cannot be instantaneous and therefore apparently healthy broilers often possess sub-clinical lesions primarily consisting of osteochondrosis and the earliest stages of BCO. Broilers with severe BCO abscesses may purposefully resist exhibiting symptoms of lameness, as a means of delaying the aggressive attacks that typically are inflicted on weak or injured birds by their flock-mates. Survivors necropsied after living for 8 weeks on wire flooring remain fully capable of walking yet they often exhibit early lesions in the femora and tibiae. Sub-clinical lesions are equally likely to develop in males and females, in left and right legs, and the status of the proximal femoral head does not determine the status of the ipsilateral or contralateral proximal tibial head and *vice versa* (Wideman et al., 2012). These observations are consistent with the interpretation that sub-clinical mechanical damage to one or more proximal leg bones need not trigger overt lameness until the damaged area becomes infected. The resulting bacterial proliferation, immunological assault by responding phagocytes (macrophages and heterophils), and widespread lysis and necrosis of the metaphyseal trabecular bone and vasculature then culminate in terminal lameness (Howlett, 1980; Duff, 1984b; Thorp et al., 1993b).

## Role of Immunosuppression

Immunosuppression caused by chicken anemia virus, infectious bursal disease virus, or environmental stressors can facilitate microbial proliferation. Immunosuppression has been implicated in the etiology of spontaneous BCO outbreaks in commercial poultry flocks (Mutalib et al., 1983a,b; Andreasen et al., 1993; McNamee et al., 1998, 1999; Butterworth, 1999; Huff et al., 2000; McNamee and Smyth, 2000). Wire flooring *per se* likely constitutes a significant stressor contributing to generalized immunosuppression and thus bacterial proliferation. For example, elevated flooring systems that deprive birds of access to floor litter stimulate chronic stress, including immunosuppression attributable to elevated blood corticosterone concentrations (El-Lethey et al., 2003). Glucocorticoid-induced femoral head necrosis has been demonstrated in adult Leghorn hens (Cui et al., 1997) and prednisolone injections caused epiphyseolysis (separation of the epiphysis from the physis) in broilers (Durairaj et al., 2012). Important roles for environmental stressors and immunosuppression clearly must be considered in investigations of the spontaneous etiology of BCO (Mutalib et al., 1983a,b; Butterworth, 1999; McNamee and Smyth, 2000). Indeed, bacterial arthritis and infection of the proximal tibiae are characteristic symptoms of turkey osteomyelitis complex (*TOC*). Environmental stressors and immunosuppression contribute to the eruption of opportunistic pathogens harbored sub-clinically in the proximal tibial joints of turkeys that develop TOC (Wyers et al., 1991; Huff et al., 1998, 1999, 2000, 2006). The involvement of many different opportunistic microorganisms suggests susceptibility to TOC may be influenced more by deficiencies in the host immune response or stress-mediated immunosuppression rather than by the pathogenicity of any one organism (Huff et al., 2000, 2005, 2006). In an experimental setting, TOC can be triggered by injecting turkey poults with repeated immunosuppressive doses of dexamethasone, a synthetic glucocorticoid (Huff et al., 1998, 1999, 2000, 2005, 2006). Stress clearly facilitates translocation and hematogenous distribution of indigenous enteric bacteria in humans (Berg, 1992).

**Figure 8:**
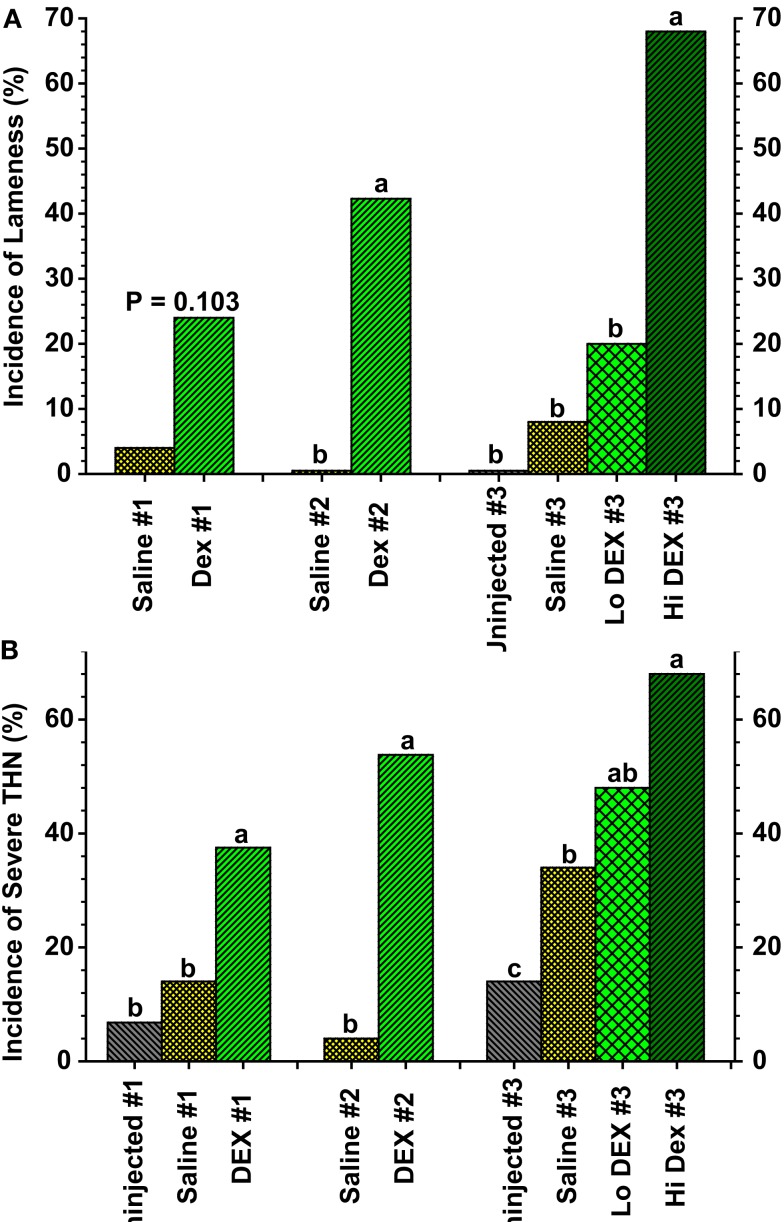
**(A)** Incidence of lameness as a percentage of the total number of birds per group for broilers in three experiments (#1 through #3) that received three (Saline #1, DEX #1) or six (Saline #2, DEX #2, Saline #3, Lo DEX #3, Hi DEX #3) injections of saline or dexamethasone (DEX). **(B)** Incidences of “severe” tibial head lesions for the uninjected and saline- or dexamethasone-injected broilers are compared in three experiments. Lesions that obviously had damaged the growth plate *per se*, or that contained caseous exudates, were recorded as being “severe.” Values reflect the percentages of all legs evaluated; within each group the data for lame birds and survivors are pooled. ^a,b,c^Values differed between the groups within an experiment (*P* < 0.05; *Z*-test; adapted from Wideman and Pevzner, 2012).

Based on the pathogenic similarities between BCO and TOC, three recent experiments were conducted to determine if dexamethasone injections might be used as a model for triggering lameness attributable to BCO in broilers. In all three experiments dexamethasone-injected broilers developed lameness primarily associated with increased incidences of BCO-like lesions of the proximal tibiae (Figure 8; Wideman and Pevzner, 2012). However, several key responses to dexamethasone in these experiments did not mirror the typical characteristics of spontaneous BCO. A major point of concern was the severe reduction in growth rates in dexamethasone-injected broilers. Growth ceased almost immediately after the initial dexamethasone injection regardless whether the birds became lame or survived. In contrast, all of the uninjected and saline-injected broilers continued growing in spite of the fact that sub-clinical BCO lesions were present in some individuals. A corresponding attenuation of body weight gain was reported for immature turkeys injected with dexamethasone (Huff et al., 1998, 1999), and for laying hens or broilers injected with glucocorticoids (Bélanger and Migicovsky, 1960; Cui et al., 1997; Durairaj et al., 2012). Even the lowest doses of dexamethasone administration severely inhibited growth while only marginally tending to increase the incidence of lameness when compared with uninjected or saline-injected controls (Wideman and Pevzner, 2012). Dexamethasone also altered the appearance of proximal femoral and tibial lesions. Epiphyseolysis revealed an apparently avascular (white) growth plate in several of the dexamethasone-injected broilers. Fatty necrosis of the tibial metaphysis was observed in dexamethasone-injected but not in saline-injected broilers (Wideman and Pevzner, 2012). A similar yellowish-colored fatty osteonecrosis of the metaphysis developed in the femur and humerus of rabbits injected with the corticosteroid methylprednisolone (Yamamoto et al., 1997). Steroid-induced FHN in Leghorn hens was attributed to adipocyte (fat cell) infiltration and an associated impairment of blood flow (Cui et al., 1997). Indeed, steroid-induced femoral head osteonecrosis in mammals is closely associated with reduced blood flow (ischemia) caused by fat emboli (lipid-loaded fibrin-platelet thrombi) that occlude the subchondral microcirculation, or by hypertrophic adipocytes that are thought to compress the vascular supply to the growth plate (Wang et al., 1977; Yamamoto et al., 1997; Boss and Misselevich, 2003). Occlusion of the tibial metaphyseal vasculature by fat micro-emboli also was observed in dexamethasone-injected broilers (Wideman and Pevzner, 2012). In contrast, the focal ischemia associated with BCO in broilers has been attributed to mechanical compression, traumatic transection by physeal clefts or abscesses, or thrombotic occlusion of metaphyseal blood vessels (Duff, 1984b, 1989a; Emslie et al., 1984; Duff and Randall, 1987; Hocking, 1992; Thorp et al., 1993b; Thorp, 1994; Wideman et al., 2012). Accordingly, the pathogenesis of lameness caused by dexamethasone does not appear to precisely recapitulate the pathogenesis of spontaneous BCO.

## Prophylactic Probiotic Treatments

**Figure 9:**
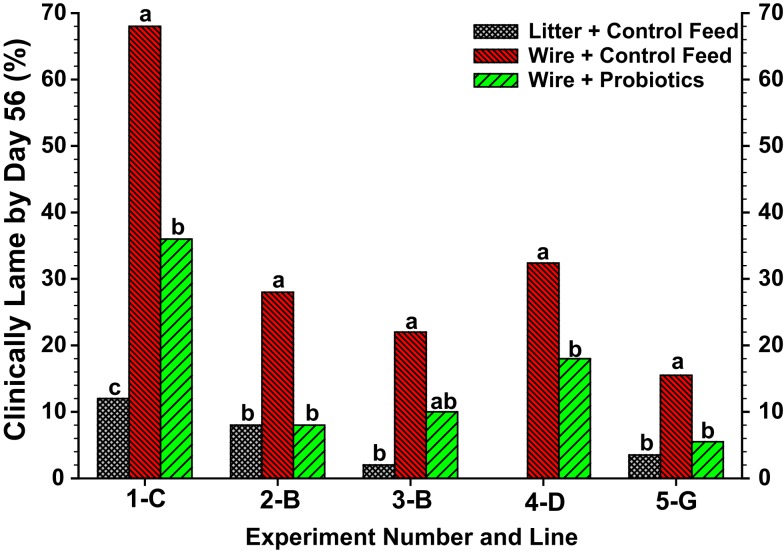
**Meta-analysis of total lameness through 56 days of age for broilers in Experiments 1–5 from Lines C, B, D and G that were reared on clean wood shavings litter and fed a control diet (litter-control feed), or reared on wire flooring and fed the control diet alone (wire-control feed) or the control diet mixed probiotics beginning on d 1 (wire-probiotics)**. ^**a,b,c**^ Values with different superscripts within an experiment differed significantly at *P* < 0.05 using repeated *Z*-tests (SigmaPlot) to compare proportions adapted from Wideman et al. (2012).

With regard to the intestinal tract as a potential site of bacterial translocation, experiments were conducted using the wire flooring model to evaluate the impact of probiotics on the incidence of lameness. In view of concerns regarding the development of antibiotic resistance in bacteria commonly associated with osteomyelitis (McNamee and Smyth, 2000; Waters et al., 2011), probiotics potentially can provide a plausible alternative for prophylactically reducing the incidence of BCO. Probiotics may interfere with the development of osteomyelitis by attenuating intestinal populations of pathogenic bacteria, improving gut health to reduce bacterial leakage (translocation) across the gut wall, or by priming the immune system to better eliminate translocated bacteria. The wire flooring model combines reduced contact with fecal material (feces accumulate beneath the wire) plus sustained re-inoculation (continuous probiotic delivery in the feed) to create ideal conditions under which a probiotic might be expected to elicit beneficial responses. Probiotics are not antibiotics and are unlikely to be effective if administered therapeutically only after lameness has developed in a flock. Indeed, probiotic administration beginning at 1 day of age, but not at 28 days of age, significantly reduced the incidence of lameness attributable to BCO in five independent experiments (Figure 9; Wideman et al., 2012). Overall, the probiotics apparently delayed the progressive deterioration of early sub-clinical lesions into the grossly degenerative abscesses that are associated with terminal lameness in broilers. These experiments indicate that bacterial translocation from the gastrointestinal tract is likely to be a significant route contributing to hematogenous infection of the bones, and that probiotics administered prophylactically (beginning at a very early age) can provide a plausible alternative to antibiotics for reducing the incidence of BCO.

## Summary and Conclusion

Bacteria enter the systemic circulation via lesions in the integument or by translocation through compromised respiratory or gastrointestinal barriers. The fenestrated endothelia of the mvp, the pvp, and the evc enable opportunistic bacteria to exit the circulatory system and infiltrate the metaphysis, physis, epiphysis, and the secondary center of ossification. The extremely rapidly growing and under-mineralized columns of chondrocytes at the proximal ends of leg bones are susceptible to micro-trauma (physeal osteochondrosis) and focal ischemia induced by torque and shear stress associated with unstable footing. Inadequate blood flow and vascular occlusion contribute to delayed mineralization and focal necrosis of the growth plate, further enhancing the structural instability and susceptibility to micro-trauma. Osteochondrotic clefts and necrotic voids provide niches for bacterial colonization outside of the surveillance of circulating leukocytes. Stress-mediated immunosuppression is permissive for bacterial translocation, enhanced hematogenous distribution, and sustained infection. We have developed a wire flooring model that causes fast growing broiler chickens to spontaneously develop high incidences of lameness attributable to pathognomonic lesions of the proximal femora and tibiae. Wire flooring imposes persistent footing instability and also constitutes a significant chronic stressor that promotes bacterial proliferation attributed to stress-mediated immunosuppression. This model for reliably triggering spontaneous osteomyelitis in large numbers of animals represents an important opportunity to conduct translational research focused on understanding the structural and functional basis of susceptibility to ischemia, epiphyseal-physeal dysfunction and necrosis, and to develop effective prophylactic and therapeutic treatments.

## Conflict of Interest Statement

The authors declare that the research was conducted in the absence of any commercial or financial relationships that could be construed as a potential conflict of interest.

## Footnotes

^1^Provisional patent No. 61/499,954 protects the exclusive rights of the University of Arkansas to all uses of the wire flooring technology within the context of evaluating or developing treatments for inducing lameness attributable to osteochondrosis, chondronecrosis, and osteomyelitis in poultry.
